# Functional and Binding H1N1pdm09-Specific Antibody Responses in Occasionally and Repeatedly Vaccinated Healthcare Workers: A Five-Year Study (2009-2014)

**DOI:** 10.3389/fimmu.2021.748281

**Published:** 2021-12-06

**Authors:** Håkon Amdam, Anders Madsen, Fan Zhou, Amit Bansal, Mai-Chi Trieu, Rebecca Jane Cox

**Affiliations:** ^1^ Influenza Centre, Department of Clinical Science, University of Bergen, Bergen, Norway; ^2^ Department of Microbiology, Haukeland University Hospital, Bergen, Norway

**Keywords:** influenza, pandemic vaccination, repeated vaccination, HI, MN, ELISA, H1N1pdm09, healthcare workers

## Abstract

**Background:**

In 2009, a novel influenza A/H1N1pdm09 emerged and caused a pandemic. This strain continued to circulate and was therefore included in the seasonal vaccines up to the 2016/2017-season. This provided a unique opportunity to study the long-term antibody responses to H1N1pdm09 in healthcare workers (HCW) with a different vaccination history.

**Methods:**

HCW at Haukeland University Hospital, Bergen, Norway were immunized with the AS03-adjuvanted H1N1pdm09 vaccine in 2009 (N=55) and divided into groups according to their vaccination history; one vaccination (N=10), two vaccinations (N=15), three vaccinations (N=5), four vaccinations (N=15) and five vaccinations (N=10). HCW are recommended for influenza vaccination to protect both themselves and their patients, but it is voluntary in Norway. Blood samples were collected pre- and at 21 days, 3, 6, and 12 months after each vaccination, or annually from 2010 HCW without vaccination. ELISA, haemagglutination inhibition (HI) and microneutralization (MN) assays were used to determine the antibody response.

**Results:**

Pandemic vaccination induced a significant increase in the H1N1-specific antibodies measured by ELISA, HI and MN. Seasonal vaccination boosted the antibody response, both in HCW with only the current vaccination and those with prior and current vaccination during 2010/11-2013/14. We observed a trend of increased antibody responses in HCW with only the current vaccination in 2013/14. A two- and three-year gap before vaccination in 2013/14 provided a more potent antibody response compared to annually vaccinated HCW.

**Conclusions:**

Our long term follow up study elucidates the antibody response in HCW with different vaccination histories. Our findings contribute to our understanding of the impact of repeated vaccination upon antibody responses.

## Introduction

Influenza is a respiratory virus that causes annual epidemics and occasional pandemics. Seasonal influenza is estimated to cause 294,000-518,000 deaths globally each year (1), but mortality can increase dramatically when a pandemic occurs. Vaccination remains the cornerstone of influenza prevention by inducing antibodies against the major surface proteins hemagglutinin (HA) and neuraminidase (NA). In Norway, annual vaccination is recommended for high-risk populations and occupational groups, including healthcare workers, (HCW) to protect the individual and patients (2).

The first pandemic of the 21^st^ century was caused by an influenza A H1N1 virus (A/California/07/2009) which was first detected in April 2009 before spreading globally (3). Norway initiated a mass vaccination campaign in October 2009, and 2.2 million people were vaccinated with the AS03-adjuvanted monovalent H1N1pdm09 vaccine. HCW were among the first to receive the vaccine to maintain the integrity of the healthcare system (4). The H1N1pdm09 strain continued to circulate as a seasonal virus after the pandemic and was included in seasonal influenza vaccines from 2010/11 to 2016/17 seasons.

Mutations in the surface glycoproteins cause antigenic drift. Annual vaccine updates are necessary to match the vaccine strains with circulating viruses, making it difficult to assess the durability of antibody response. The immune response to influenza is multifaceted and shaped by several factors, including previous influenza infections or vaccinations, age and health conditions (5). Despite decades of use, the impact of annual repeated influenza vaccination on antibody responses remains unclear, with conflicting results reported with either increases or decreases in antibody responses (6, 7). Furthermore, the vaccine coverage among recommended high-risk groups and HCW in Norway is far from WHO´s goal of 75% (8). As a result, many HCW are not annually vaccinated, and their vaccination status varies.

We conducted a five-year follow up of HCW vaccinated with the AS03-adjuvanted pandemic vaccine in 2009, followed by seasonal trivalent inactivated influenza vaccines (IIV) during the following years (9). The H1N1pdm09 component (A/California/07/2009) of the vaccine remained unchanged during the five-year period, giving us the unique opportunity to investigate the long-term H1N1pdm09 antibody response in groups of HCW categorized based on their annual vaccination history over the five-year period.

## Materials and Methods

### Clinical Trial

Fifty-five participants were selected from a clinical trial of HCW vaccinated in 2009 with the AS03-adjuvanted monovalent pandemic split H1N1 virus vaccine containing the A/California/07/2009 (H1N1pdm09) virus [Pandemrix, GlaxoSmithKline (GSK), Belgium] at Haukeland University Hospital, Bergen, Norway. The participants were followed up for a five-year period and were selected and grouped based on the number of vaccinations during the study period. Ten HCW received solely the pandemic vaccine, fifteen HCW received the pandemic vaccine and one additional IIV, five HCW received the pandemic vaccine and two additional IIV and fifteen HCW received the pandemic vaccine and three IIV. Ten HCW received four consecutive IIV, in addition to the pandemic vaccine. The trivalent seasonal inactivated influenza vaccine [IIV, either sub-unit (Influvac, Abbott Laboratories) or split-virion (Vaxigrip, Sanofi Pasteur)] was used from 2010/10 to 2013/14 containing the A/California/07/2009 (H1N1pdm09) virus as the A/H1N1 component.

All HCW provided written informed consent before inclusion in the study (10). The study was approved by the regional ethics committee (Regional Committee for Medical Research Ethics, Western Norway (REK Vest 2012/1772) and the Norwegian Medicines Agency. The trial is registered in the European Clinical Trials Database (2009-016456-43), and National Institute for Health Database Clinical trials.gov (NCT01003288).

### Sampling

Blood samples were collected pre-pandemic vaccination (day 0), at 21 days, and 3, 6, 12, 24, 36, 48 and 60 months after pandemic vaccination. HCW who were annually vaccinated provided additional blood samples at 21 days, 3, and 6 months after each vaccination. Serum samples were stored at -80°C until analysed.

### HA Proteins and Influenza Viruses

Whole H1 HA (trimeric A/California/04/09) was generated using the baculovirus expression system for the ELISA (11). Recombinant baculoviruses were passaged three times through Sf9 cells, before infection of High-five cells. Purified proteins were analysed using sodium dodecyl sulphate polyacrylamide gel electrophoresis (SDS-PAGE) and quantified by infrared spectrometer (DirectDetect^®^, Milipore Corporation). For the HI assay, A/California/07/2009 (H1N1) virus was beta-propiolactone (BPL) inactivated (Influenza Reagents Resources). For the MN assay, a recombinant A/California/07/2009 (H1N1) virus (X-179A) was propagated in-house in 10-day-old embryonated hen´s eggs and frozen at -80°C until used.

### ELISA

HA specific serum IgG antibodies were measured by indirect enzyme-linked immunosorbent assay (ELISA), as previously described (12). Ninety-six-well plates (Invitrogen) were incubated with HA protein overnight (1 μg/ml in PBS). The next day, five-fold serial dilutions of serum were added, followed by one hour incubation at 37°C. Horseradish peroxidase (HRP)-conjugated goat anti-human IgG (BD Biosciences, USA, 555788) were added to the plates, and detected with colorimetric substrate [3,3´,5,5´-3,3´,5,5´-tetramethylbenzidine (TMB) (BD Biosciences, USA)]. Absorbances were measured with a microplate reader (Bio-Tek) at the optical density (OD) of 450nm. ELISA endpoint titres were defined as the reciprocal of the highest dilution of serum to give a detectable measurement (OD value over 3 standard deviations above the mean of blank controls).

### Hemagglutination Inhibition Assay

Antibodies directed towards the receptor binding site of HA were analysed using the hemagglutination inhibition (HI) assay, as previously described (10). Sera was heat-inactivated and treated with receptor destroying enzyme (Denka Seiken, Japan). Eight HA units of the A/California/07/2009 (H1N1pdm09) virus were added to 2-fold serial dilutions of RDE treated sera in phosphate buffered saline (PBS) for one hour before incubation with 0.7% turkey red blood cells for 30 minutes. The HI titre was defined as the reciprocal of the highest dilution of serum that inhibited 50% hemagglutination. Negative values were assigned a value of 5 for calculation purposes.

### Microneutralization Assay

The microneutralization (MN) assay was used for measuring neutralizing antibodies as previously described (13). Sera were heat-inactivated at 56°C for 30 minutes and added in 3-fold serial dilutions to a ninety-six-well cell culture plate (Nunclone Delta surface, USA), with H1N1pdm09 like-virus (X179a, 2000 TCID50/ml). Madin-Darby Canine Kidney (MDCK) cells were added after one hour incubation in room temperature, and the plates were then incubated in 37°C for 18 hours. The next day, cells were fixed with hydrogenperoxide (Sigma-Aldrich, USA, H-1009) in methanol (Sigma-Aldrich, USA, 32213) (0.6% H_2_O_2_) in 20 minutes. Mouse anti-influenza A nucleoprotein antibodies (AbD Serotec, USA, MCA400) was added, and the plates were incubated for one hour at 37°C. HRP secondary antibody (Dako, Denmark, P0260) was added followed by one-hour incubation. Influenza virus was detected using TMB, before reading at 450 and 620nm to calculate the OD-value. The dilution of serum that provided 50% inhibition of infection was calculated as the MN titre. Negative values were assigned a value of 5 for calculation purposes.

### Statistical Analysis

The appropriate statistical tests were used to detect differences within and between the different groups. All analysis were conducted in GraphPad Prism version 8.0 for Mac, (GraphPad Software, USA). Correlations between the assays are presented as Pearson´s *r*, alpha = 0.05. A p-value <0.05 were considered statistically significant in all analysis.

## Results

### Demographics of the Healthcare Workers

Pandemic and seasonal influenza vaccination were recommended for all HCW but voluntary and provided free of charge by the hospital. Fifty-five HCW received the AS03-adjuvanted H1N1pdm09 pandemic vaccine in 2009. During the subsequent seasons from 2010/11 - 2013/14, IIV containing the same H1N1pdm09 virus (A/California/07/2009) was used. HCW were retrospectively divided into different groups according to their number of seasonal influenza vaccinations and vaccination history during the study period (**Table 1**). Most of the HCW were female (84%) corresponding to the gender distribution of Norwegian healthcare workers. The majority of the HCW (64%) worked clinically and had a history of previous influenza vaccination (75%) The mean age of the study population was 37 years old.

**Table 1:** Demographics and vaccination histories of the healthcare workers.

Number of vaccinations (n)	One vaccination (10)	Two vaccinations (15)	Three vaccinations (5)	Four vaccinations (15)	Five vaccinations (10)
**Age (mean)**	41	40	27	38	37
**Sex (female/male)**	8/2	13/2	3/2	12/3	10/0
**Clinical work (yes/no)**	2/8	11/15	4/1	11/4	7/3
**Vaccination status 2009 - 2013 (V/N) ^a,b^**	V-N-N-N-N (10)	V-V-N-N-N (6)	V-V-N-N-V (5)	V-V-V-V-N (6)	V-V-V-V-V (10)
		V-N-N-N-V (5)		V-V-V-N-V (4)	
		V-N-V-N-N (4)		V-V-N-V-V (5)	
**Year of vaccination (n)**	2009 (10)	2009 (15)	2009 (5)	2009 (15)	2009 (10)
		2010 (10)	2010 (5)	2010 (15)	2010 (10)
		2011 (4)	2011 (0)	2011 (10)	2011 (10)
		2012 (0)	2012 (0)	2012 (11)	2012 (10)
		2013 (5)	2013 (5)	2013 (9)	2013 (10).
**Previous influenza vaccination (yes/no)**	7/3	11/4	2/3	12/3	9/1

a V, vaccinated; N, Not vaccinated.

b We had no virological surveillance of the healthcare workers.

### Pandemic Vaccination Induced a Strong Increase in Antibodies

**Figure 1:**
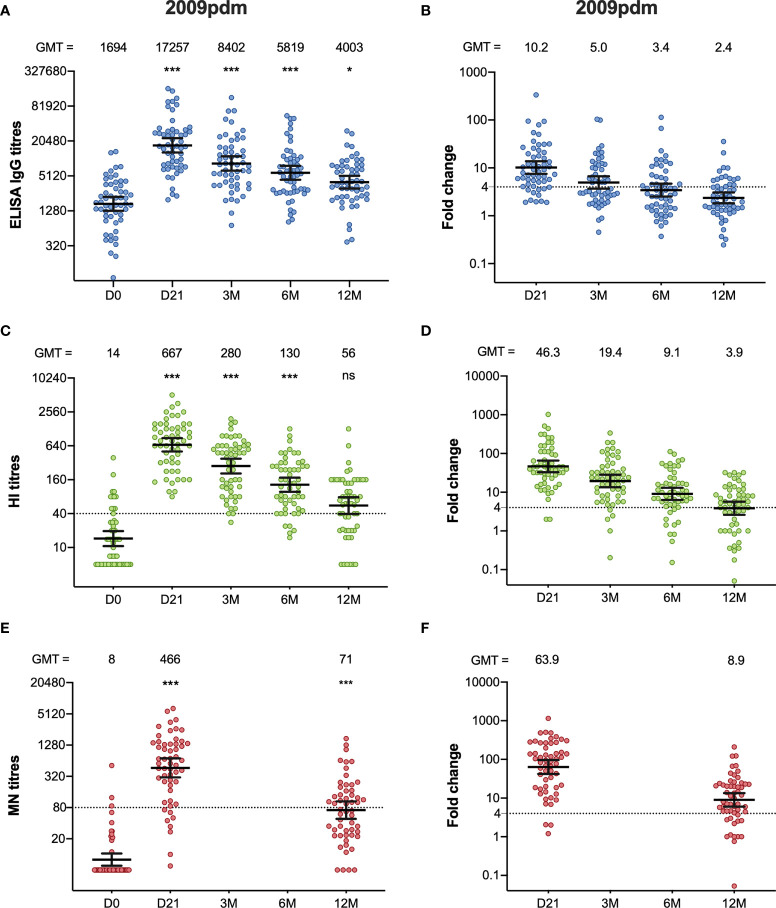
The H1N1pdm09-specific ELISA IgG, HI, and MN antibody response after pandemic vaccination. The ELISA IgG **(A)**, HI **(C)** and MN-titres **(E)** measured in the HCW during 2009/10 (2009pdm). Each symbol represents an individual ELISA, HI and MN titre, and the horizontal line representing the geometric mean titre (GMT) and 95% confidence interval. The GMT for each timepoint in each assay is shown over the graph. The dotted line at 40 and 80 represents the protective titre, in HI and MN respectively. The fold-changes from pre-vaccination ELISA IgG **(B)**, HI **(D)** and MN titres **(F)** measured from pre-vaccination titres (D0) to post- pandemic vaccination (D21, 3M, 6M, 12M) are shown. Each symbol represents an individual ELISA IgG, HI and MN fold-change from pre-vaccination titres to day 21, 3, 6 and 12 months after vaccination. The horizontal line shows the geometric mean with 95% confidence interval. The dotted line at 4 indicates seroconversion, and the GMT is shown above the graph. Statistical differences were tested using the Friedman test, with Dunn ´s multiple comparison test. The stars indicate significant differences from pre-vaccination titres, using D0 as a reference. ***P < 0.001, *P < 0.05. Ns, not significant.

We measured the IgG-specific antibodies binding to full length HA H1N1pdm09 (A/California/07/2009) in ELISA (**Figures 1A, B**). H1N1pdm09 specific binding antibodies were detected in all HCW (55/55) before pandemic vaccination, although at low levels. Pandemic vaccination significantly increased the IgG titres at day 21, 3, 6 and 12 months after vaccination (p < 0.05), with a fold change of 10.2. The titres were significantly higher than pre-vaccination titres at 3, 6 and 12 months after vaccination, despite the titres gradually waned from 21 days after vaccination.

We used HI to measure the functionality of the antibodies (**Figures 1C, D**). An HI titre ≧ 40 is considered as protective, with a 50% reduction in the risk of contracting influenza (14). Twelve of the 55 of the HCW had an HI titre ≥40 before pandemic vaccination. Vaccination induced a significant increase in HI titres measured at 21 days, 3 months and 6 months after vaccination (p <0.001). All HCW had an HI titre ≥40 at 21 days after vaccination, and the fold-change was 46.3. The HI titres waned during the 2009/10-season, but the vaccine fulfilled the criteria of the European Committee for Medicinal Products for Human Use (CHMP), which were pre- and post-vaccination geometric mean ratio >2.5, seroconversion rate >40%, and seroprotection rate >70%, for up to 6 months (10). Two-thirds (37/55) of the HCW had HI titres ≥40 at 12 months.

We further measured the neutralising antibodies using the MN assay, which detects neutralising antibodies that prevent virus infection in cell culture (**Figures 1E, F**). Only three HCW (3/55) had MN titres >80 before pandemic vaccination, which has been suggested to be seroprotective (15). Forty-eight of fifty-five HCW (87%) had titres >80 measured at 21 days after pandemic vaccination, and the titres increased significantly compared to pre-vaccination titres, (p < 0.001) with a fold-change of 63.9. The titres decreased from 21 days after vaccination from 12 months after vaccination but were significantly higher compared to pre-vaccination titres (p < 0.001).

Ten of the HCW received only the pandemic vaccination during the five-year period, but annual serum samples were collected prior to each influenza season. Interestingly, five years after pandemic vaccination, 60% of HCW (6/10) had protective HI titres (≥40) and 50% (5/10) had a MN titre >80 (**Supplementary Figure 1**). None of these HCW who had protective HI or MN titres five years after pandemic vaccination had protective titres pre-pandemic vaccination.

### Seasonal Vaccination Boosted the Antibody Response, Regardless of Prior Vaccination

We grouped the HCW according to their vaccination status during the different seasons into three different groups: unvaccinated, prior and current vaccination, or current vaccination only. Since all HCW received the pandemic vaccine, we had no HCW who only received the current vaccine in 2010/11.

**Figure 2:**
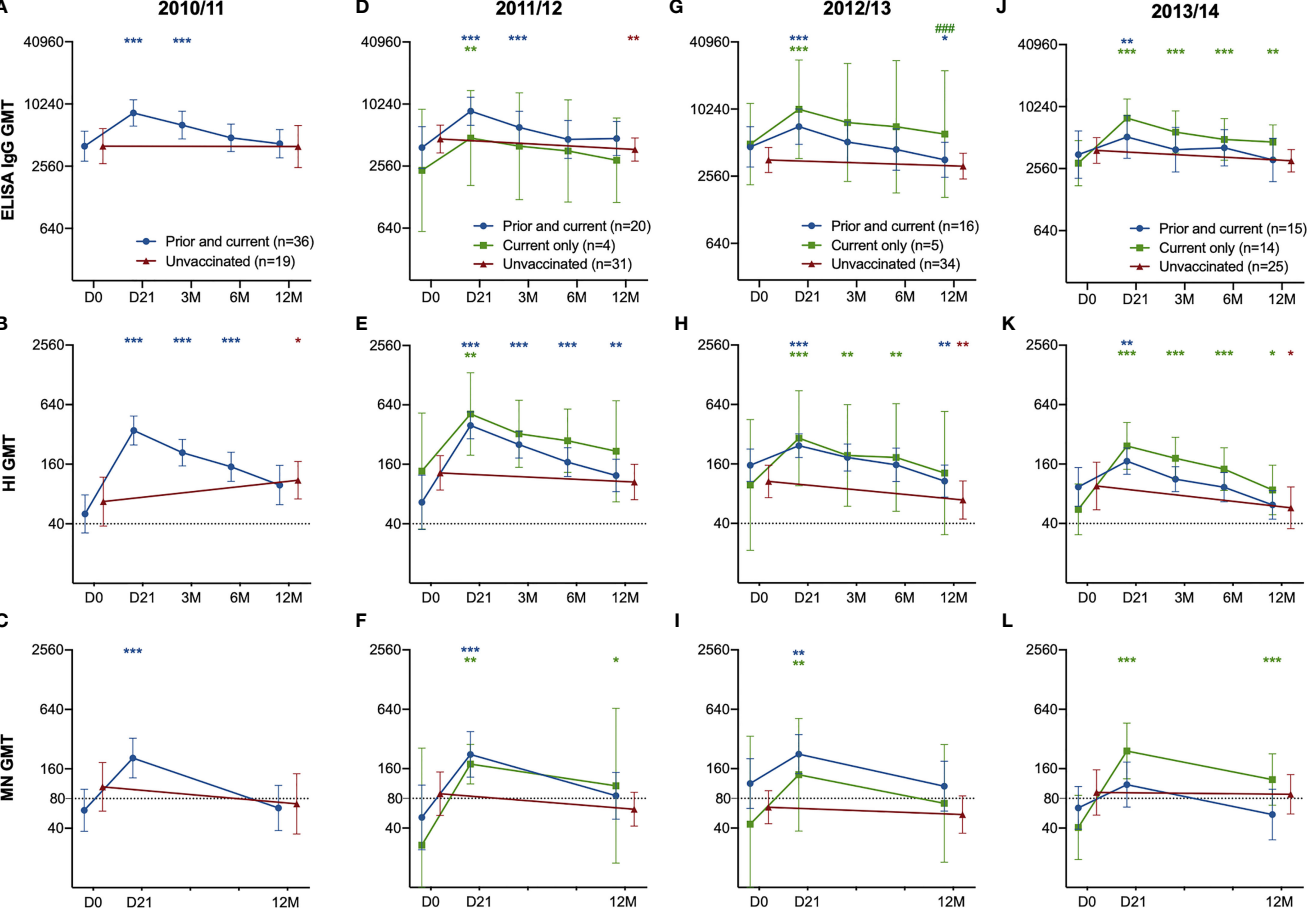
The H1N1pdm09-specific ELISA IgG, HI and MN antibody response in HCW with the prior and current vaccination, current vaccination only and unvaccinated HCW. The ELISA IgG **(A)**, HI **(B)** and MN-titres **(C)** in HCW who received the prior & current vaccination in 2010/11 compared to unvaccinated HCW in 2010/11. The ELISA IgG **(D, G, J)**, HI **(B, E, H, K)** and MN titres **(C, F, I, L)** in HCW who received the prior & current vaccination in 2011/12 – 2013/14 compared to HCW who received only the current vaccination, and unvaccinated HCW. The geometric mean titres with 95% confidence interval are presented. The dotted line at 40 and 80 represents the protective titre, in HI and MN respectively. The data was log-transformed and analysed with the two-way ANOVA with Dunnetts test for multiple comparisons, for detecting differences within a group. Statistical differences between the groups were tested with linear mixed-effect models, with Dunnett´s test for multiple comparisons. The stars indicate significant increases within a group, with pre-vaccination titres (D0) as the reference timepoint. ***P < 0.001, **P < 0.01, *P < 0.05. ^###^ Indicates statistical differences (P<0.05) between groups.

**Figure 3:**
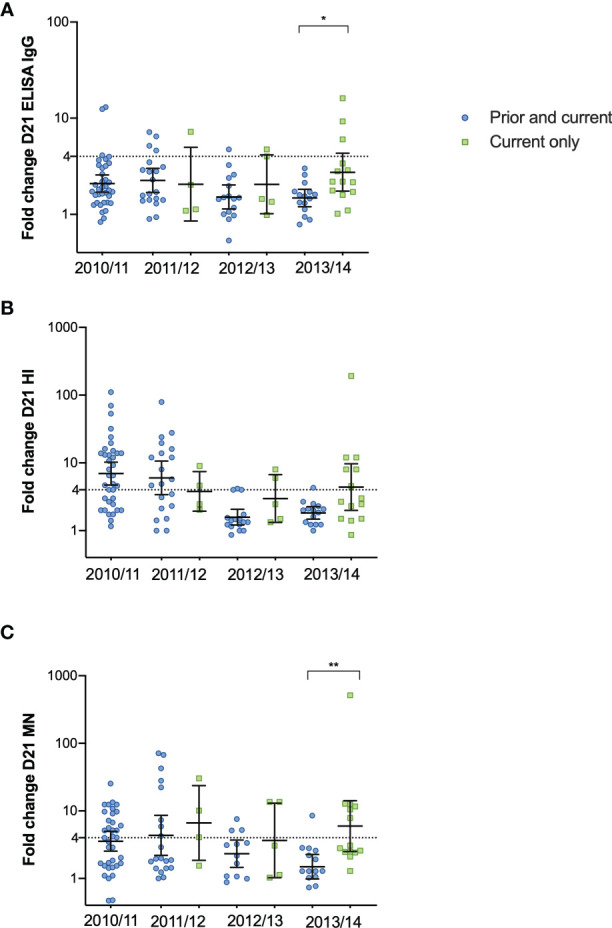
The fold change after vaccination in 2010/11 – 2013/14 in HCW receiving the prior and current vaccination and the current vaccination only. The fold-change from pre-vaccination titres (D0) to post-vaccination titres (D21) measured in ELISA **(A)**, HI **(B)** and MN titres **(C)** in the HCW with the current vaccination only and those with the prior and current vaccination. Each symbol represents an individual ELISA, HI and MN fold change. The horizontal line shows the geometric mean with 95% confidence interval, and the dotted line at 4 indicates seroconversion. Statistical differences between the groups were tested with the Kruskal-Wallis test, with Dunn´s multiple comparisons test. **P < 0.01, *P < 0.05.

Seasonal vaccinations significantly boosted both the quantity (ELISA) and quality (HI and MN) of the antibody response each year, regardless of prior vaccination status (**Figures 2A–L**). The only significant difference among those vaccinated was measured in ELISA in 2012/2013, where HCW who had not taken influenza vaccination in the prior season had significantly higher titres 12 months after vaccination compared to HCW vaccinated in both seasons (**Figure 2C**). We observed no differences in antibody responses during the seasons 2011/12 – 2012/13 between the groups, but HCW vaccinated only in the current 2013/14 season had a trend of a better antibody response compared to those with the prior vaccination. (**Figures 2D, H, L**). HCW without prior vaccination had significantly higher GMT (Geometric mean titres) 12 months after vaccination in 2013/14 compared to pre-vaccination GMT (**Figures 2J, K, L**). Compared to those who had been vaccinated in 2012/13 and received the current vaccination in 2013/14, the HCW without the prior vaccination had a significantly higher fold-change 21 days after vaccination in the 2013/14 season measured in ELISA and MN (p < 0.05) (**Figures 3A, C**).

Each season, HCW who were vaccinated had significant increases in their antibody titres measured at day 21 after vaccination in all assays (p < 0.05) (**Figures 2D–L**), except in the MN titres of the HCW with both prior and current vaccination at 21 days post-vaccination 2013/14. (**Figures 2L**). Also, the unvaccinated HCW showed a significant increase in HI titres in 2011/12, probably due to infection (**Figure 2B**). Antibodies decreased over time in subsequent years in the unvaccinated HCW (**Figure 2D–L**).

### Time Duration Between Vaccinations Impacted the Fold Change After Vaccination in 2013

**Figure 4:**
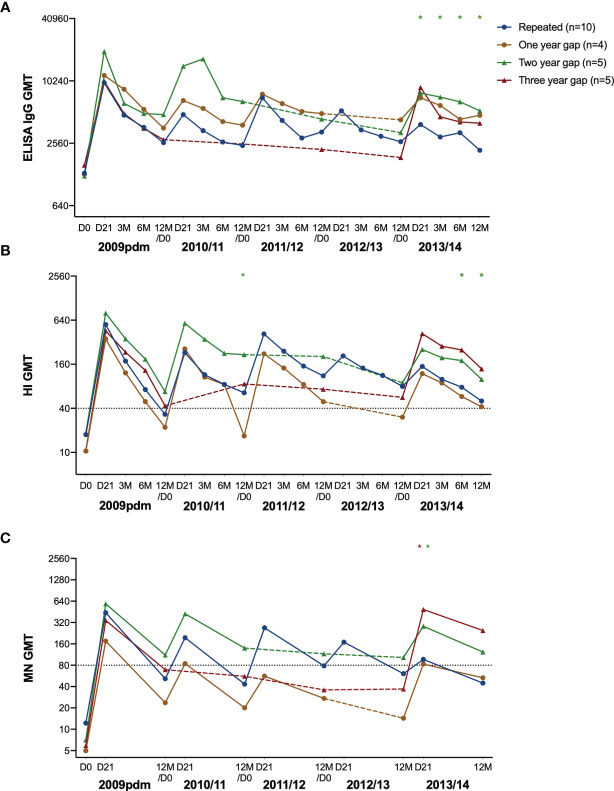
The H1N1pdm09-specific antibody response over five years after pandemic and seasonal vaccinations. The geometric mean titre with 95% confidence interval measured in ELISA **(A)**, HI **(B)**, and MN **(C)** in repeatedly vaccinated HCW, and HCW with a one-, two- and three-year gap in vaccinations before vaccination in 2013/14. The titres at pre-vaccination (D0), and at 21 days (D21), 3, 6, and 12 months after vaccination are shown. The gap-years without vaccination is illustrated with a dotted line. The data was log-transformed and compared between groups with linear mixed-effect models, with Dunnett’s test for multiple comparisons. The repeatedly vaccinated HCW was used as the reference group. ***P < 0.001, **P < 0.01, *P < 0.05.

We further investigated the antibody responses in HCW with repeated annual vaccination compared to those with one-, two- or three-year gap in vaccination before vaccination in 2013. Antibodies increased after vaccination in all years but with some variations between the subgroups (**Figures 4A–C**). Antibodies declined over time in HCW who choose not to be vaccinated with seasonal vaccine, except in the HI titres in 12M 2009/10 to 12M 2010/11 in the three-year gap group (**Figures 4B**). Interestingly, the group with a two-year gap between vaccinations in 2010/11 and 2013/14 had consistently high antibody titres and had seroprotective HI- and MN antibodies at 12M 2012/13, two years after their last vaccination (**Figures 4B, C**).

When comparing the subgroups against the annually repeated group, the group with a two-year gap had significantly higher titres at day 21, 3, 6 and 12 months after vaccination in 2013/14 measured by ELISA (**Figure 4A**), and HI at 3 months and 6 months (**Figure 4B**). In addition, they had significantly higher MN titres 21 days after vaccination in 2013/14 (**Figure 4C**). The three-year gap group had significantly higher titres compared to the repeated group at 21 days after vaccination in 2013/14.

**Figure 5:**
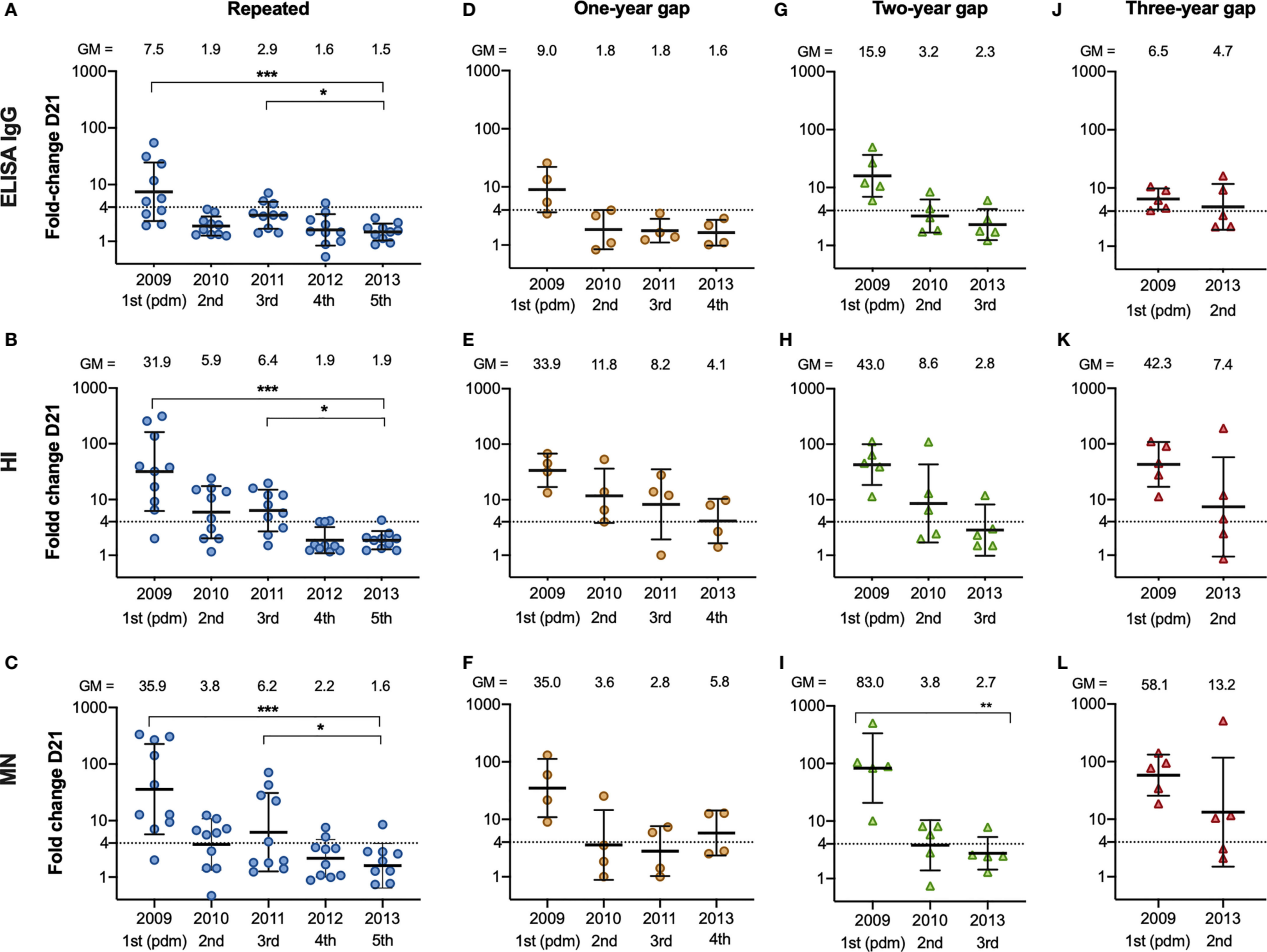
The fold change after vaccination from 2009/10 – 2013/14 in repeatedly vaccinated and HCW with a one-, two- and three-year gap in vaccinations before vaccination in 2013/14. The fold change from pre-vaccination titres (D0) to post-vaccination titres (D21) measured in ELISA **(A)**, HI **(B)** and MN **(C)** in the HCW with repeated vaccinations, one-year gap **(D–F)** two-year gap **(G–I)** and a three-year gap **(J–L)** in vaccinations before getting vaccinated in 2013/14. Each symbol represents an individual ELISA, HI and MN fold change. The horizontal line shows the geometric mean with geometric standard deviation. The geometric mean is shown above the graph, and the number of vaccinations is shown below the graph. The dotted line at 4 indicates seroconversion. The Friedman test was used for detecting differences within the different subgroups, with Dunn´s test for multiple comparisons and the fold change in 2013/14 as the reference timepoint. ***P < 0.001, **P < 0.01, *P < 0.05.

We assessed the fold changes in the repeated group, and the groups with one-, two, or three-year gap in vaccination before vaccination in 2013 (**Figure 5**). We observed lower antibody fold changes in the repeated group after vaccination in 2012/13 and 2013/14, in all assays (5A, E, I), which were their fourth and fifth vaccinations (5A, B, C). We further analysed the correlation between the different serological assays using all antibody titres (**Supplementary Figure 2**) and all correlations were statistically significant in the Pearson correlation test. The correlation coefficient was highest between the HI and MN titres (0.73) compared to 0.57 between the HI and ELISA titres, and 0.54 between the MN and ELISA titres.

## Discussion

HCW have a higher risk of influenza infections due to occupational exposure and are an important target group for influenza vaccination (16). However, the influenza vaccine coverage among HCW in Europe is often low (17). Current IIV offers suboptimal protection but remains the best option to prevent the burden of influenza. Despite IIV being used for decades, there are still unanswered questions regarding the durability of antibody responses and vaccine effectiveness after repeated vaccination. Several studies have shown that repeated vaccination impairs the antibody response (18, 19), while others have found no adverse effect on the antibody response after repeated vaccination (7, 20). The H1N1pdm09 virus from 2009 (A/California/07/2009) was included in seasonal vaccines up to the 2016/2017-season, providing a unique opportunity to study the long-term antibody response to H1N1pdm09 without the complication of strains updates. This five-year study provides insight into antibody responses after pandemic and seasonal vaccinations in HCW with different influenza vaccination histories. Our study elucidates the impact of an AS03-adjuvanted influenza vaccine, and how the vaccination history shapes the antibody response.

The pandemic vaccine induced a potent and durable antibody response. We have previously shown durable HI antibodies in HCW receiving only the pandemic vaccine (9) and we have extended these findings to show that 50-60% of HCW with only pandemic vaccination (n = 10) had protective HI (6/10) and MN (5/10) titres 5 years post-vaccination. Although these unvaccinated HCW had a significant increase in HI titres by the end of the season 2010/11, which could be due to infection, young adults between 20-39 years old had the highest influenza attack rate in Norway in 2010/11 (21, 22). The persistence of antibodies in the HCW with protective titres up to five years after pandemic vaccination was caused by the AS03 adjuvant, which has been shown to induce higher T-and B-cell responses than non-adjuvanted vaccines (23), by activation of more naïve and memory B-cells (24). Others have also found that the AS03-adjuvanted pandemic vaccine was highly immunogenic (23, 25), and superior to non-adjuvanted monovalent H1N1pdm09 vaccines (26). The durability of these antibodies induced after adjuvanted vaccination shows the importance of inclusion of an adjuvant, which could be used in pandemic vaccine development for other possible respiratory virus pandemic threats.

All HCW received the pandemic vaccination, but their seasonal vaccination status varied during the following years. We grouped the HCW to see the differences between the unvaccinated HCW, to those who only received the current vaccination, and those who had received both the previous and current seasonal vaccination. Some studies have reported that prior vaccination can attenuate the antibody response following the current seasonal vaccination (7, 27). We observed no differences between the groups in 2011/12 – 2012/13, but the groups varied in sample size. The groups had similar sample sizes in 2013/14 and we found a trend (although not significant) of a superior antibody response in the group with the current vaccination only compared to those who had previously received seasonal vaccination. Similarly, a recent study found the lowest influenza A/H1N1pdm09 positivity rate in every influenza-season between 2012/13 – 2017/18 in individuals with current vaccination only compared to prior vaccination only or current and prior vaccination (28).

HCW with five subsequent vaccinations had a reduced boosting effect post-vaccination in 2012/2013 and 2013/14, after their fourth and fifth vaccination with the same H1N1pdm09 strain, compared with their third vaccination. This suggests that the fold change after vaccination is reduced after the third vaccination against the same A/California/07/2009 (H1N1pdm09) antigen. Conversely, a gap-year between vaccinations is beneficial in terms of antibody boosting to the same strain with fold changes >4 in HI and MN observed in 2013/14 after their fourth vaccination. We observed that the group with a three-year gap between their two vaccinations (vaccinated in 2009/10 and 2013/14) had the highest fold change after vaccination in 2013/14 compared to the other groups. However, subject numbers were low in the groups of a two-year gap and a three-year gap before vaccination in 2013/14, so the results should be interpreted with caution. Importantly the HCW were not optimally protected during the years without vaccination and had a higher risk of contracting influenza A/H3N2 or B during this time. Therefore, annual vaccination is favourable despite the probability of a reduced boosting of the antibody titres, as we observed in the repeatedly vaccinated HCW.

A recent study assessed different mathematical models to explain the difference in antibody-boosting in individuals who skipped vaccination for at least three years, compared to repeatedly vaccinated individuals, and found an increased boost in those who skipped vaccinations (29). We observed a reduced boosting in repeatedly vaccinated HCW in 2013/14, which could be a explained by a more rapid clearance of vaccine antigen in individuals with higher baseline titres (30), shortening the germinal center reaction (31). Furthermore, pre-existing antibodies may bind to and mask epitopes in the vaccine antigens, which would limit stimulation and expansion of B-cells (29). Our results agree with previous studies (18, 32) and suggest that the antibody response against the same antigen is diminished following repeated vaccination.

It has been known for decades that the antibody response following seasonal influenza vaccination is shaped by previous influenza encounters (33), either from infection or by vaccination. The “antibody ceiling” effect that we observed in the repeatedly vaccinated HCW has been previously reported in individuals with repeated influenza vaccinations (30, 32, 34), and we found a reduced antibody boosting after vaccination in the repeated group after their fourth and fifth vaccination. The “antibody ceiling” effect has been observed in individuals with high pre-existing titres (34) and may be due to antibody focusing to conserved epitopes on the HA. Although a reduced boosting in antibodies is observed in repeatedly vaccinated individuals, unvaccinated individuals have a higher risk of influenza infection due to the lack of humoral and cellular immunity to circulating strains (28). Further studies that investigates the impact of HA-specific antibodies and T-cells in repeatedly vaccinated individuals upon clinical protection are needed. The use of an adjuvanted vaccine was favourable in terms of priming and maintaining antibodies in our cohort, and perhaps adjuvanted influenza vaccines should be considered when vaccinating individuals that are repeatedly vaccinated, such as HCW and elderly. In future studies, vaccine effectiveness and antibody response of repeatedly vaccinated individuals after receiving an adjuvanted seasonal influenza vaccine should be investigated to see if that could overcome the “antibody ceiling” effect.

Caveats to our study should be considered. The numbers of HCW in the different subgroups were limited, and most were female. Our results cannot be generalized to all influenza seasons due to antigenic drift allowing the virus to escape host immunity and subsequent need for influenza vaccine updates. Since the adjuvanted pandemic vaccine was highly immunogenic, the antibody response may differ in other populations where the first vaccination was a non-adjuvanted vaccine. Although we asked participants if they had experienced influenza like illness, we did not perform virological surveillance during the study period, so we cannot exclude natural influenza infection that may have boosted the antibody responses. However, the main strength of our study is long term follow up with blood samples over a five-year period and the use of three serological assays (ELISA, HI and MN) which complement the limited number of other similar studies (35, 36).

In summary, our findings provide insight into the antibody responses in HCW with different vaccination statuses over a five-year period after pandemic and seasonal influenza vaccinations. We found that the adjuvanted pandemic vaccine elicited a robust antibody response, and HCW with only the current vaccination and with 2- and 3 gap-years before vaccination in 2013/14 had a better antibody response compared to repeatedly vaccinated HCW. However, seasonal vaccinations are the cornerstone of protection, and without vaccination HCW are more likely to be infected with circulating viruses, increasing the potential risk of infecting their patients. Our study supports the policy of annual vaccination to provide optimal protection for each influenza season, and it contributes to our understanding of the antibody response following repeated vaccination.

## Data Availability Statement

The raw data will be made available upon reasonable request to the corresponding author.

## Ethics Statement

The studies involving human participants were reviewed and approved by Regional Ethics Committee (REKVest-2012/1772), and the Norwegian Medicines Agency (Clinicaltrials.gov NCT01003288). The participants provided their written consent to participate in this study.

## Author Contributions

RC and M-CT designed the study. HA, M-CT, FZ, and AM conducted the laboratory analysis. HA, M-CT and AB conducted the statistical analysis. HA and RC analysed the data and wrote the manuscript. All authors have read the final version of the manuscript. All authors contributed to the article and approved the submitted version.

## Funding

This work was supported by intramural funding by the Influenza Centre at the University of Bergen. The Influenza Centre is funded by the University of Bergen, Ministry of Health and Care Services Norway (F-11628), the Trond Mohn foundation (TMS2020TMT05), the European Union (EU IMI115672 FLUCOP, H2020 877866 INCENTIVE and H2020 101037867 VACCELERATE, EU IMI101007799 Inno4Vac), Nanomedicines Flunanoair (ERA-NETet EuroNanoMed2, JTC2016) and the Research Council of Norway Globvac program (284930).

## Conflict of Interest

The authors declare that the research was conducted in the absence of any commercial or financial relationships that could be construed as a potential conflict of interest.

## Publisher’s Note

All claims expressed in this article are solely those of the authors and do not necessarily represent those of their affiliated organizations, or those of the publisher, the editors and the reviewers. Any product that may be evaluated in this article, or claim that may be made by its manufacturer, is not guaranteed or endorsed by the publisher.

## References

[1] Paget J Spreeuwenberg P Charu V Taylor RJ Iuliano AD Bresee J. Global Mortality Associated With Seasonal Influenza Epidemics: New Burden Estimates and Predictors From the GLaMOR Project. J Glob Health (2019) 9(2):20421. doi: 10.7189/jogh.09.020421 PMC6815659 31673337

[2] Poland GA Tosh P Jacobson RM. Requiring Influenza Vaccination for Health Care Workers: Seven Truths We Must Accept. Vaccine (2005) 23:2251–5. doi: 10.1016/j.vaccine.2005.01.043 15755605

[3] World Health Organization. Pandemic (H1N1) 2009 – Update 68. Available at: http://www.who.int/csr/don/2009_10_02/en/.

[4] Helse og omsorgsdepartementet. Beredskap Mot Pandemisk Influensa Meld. St. 16 (2012–2013). Available at: https://www.regjeringen.no/contentassets/a7c7e93dbe8f44d2a8fe892768e429c5/no/pdfs/stm201220130016000dddpdfs.pdf.

[5] Castrucci MR. Factors Affecting Immune Responses to the Influenza Vaccine. Hum Vaccin Immunother (2018) 14(3):637–46. doi: 10.1080/21645515.2017.1338547 PMC5861809 28617077

[6] Belongia EA Skowronski DM McLean HQ Chambers C Sundaram ME De Serres G. Repeated Annual Influenza Vaccination and Vaccine Effectiveness: Review of Evidence. Expert Rev Vaccines (2017) 16:7. doi: 10.1080/14760584.2017.1334554 28562111

[7] Beyer WEP de Bruijn IA Palache AM Westendorp RGJ Osterhaus ADME. Protection Against Influenza After Annually Repeated Vaccination: A Meta-Analysis of Serologic and Field Studies. Arch Intern Med (1999) 159(2):182–8. doi: 10.1001/archinte.159.2.182 9927102

[8] Folkehelseinstituttet. Influenza Vaccination Coverage in Norway 2018/2019 Season (2019). Available at: https://www.fhi.no/sv/influensa/influensavaksine/vaksinasjonsdekningstall-for-influensavaksine/.

[9] Trieu MC Jul-Larsen Å Sævik M Madsen A Nøstebakken JK Zhou F. Antibody Responses to Influenza A/H1N1pdm09 Virus After Pandemic and Seasonal Influenza Vaccination in Healthcare Workers: A 5-Year Follow-Up Study. Clin Infect Dis (2019) 68(3):382–92. doi: 10.1093/cid/ciy487 PMC6336911 29893797

[10] Madhun AS Akselsen PE Sjursen H Pedersen G Svindland S Nøstbakken JK. An Adjuvanted Pandemic Influenza H1N1 Vaccine Provides Early and Long Term Protection in Healthcare Workers. Vaccine (2010) 29:266–73. doi: 10.1016/j.vaccine.2010.10.038 21034828

[11] Krammer F Pica N Hai R Margine I Palese P. Chimeric Hemagglutinin Influenza Virus Vaccine Constructs Elicit Broadly Protective Stalk-Specific Antibodies. J Virol (2013) 87(12):6542–50. doi: 10.1128/JVI.00641-13 PMC3676110 23576508

[12] Tete SM Krammer F Lartey S Bredholt G Wood J Skrede S. Dissecting the Hemagglutinin Head and Stalk-Specific IgG Antibody Response in Healthcare Workers Following Pandemic H1N1 Vaccination. NPJ Vaccines (2016) 1:16001. doi: 10.1038/npjvaccines.2016.1 29250435 PMC5707877

[13] Rowe T Abernathy RA Hu-Primmer J Thompson WW Lu X Lim W. Detection of Antibody to Avian Influenza A (H5N1) Virus in Human Serum by Using a Combination of Serologic Assays. J Clin Microbiol (1999) 37(4):937–43. doi: 10.1128/JCM.37.4.937-943.1999 PMC88628 10074505

[14] Hobson D Curry RL Beare AS Ward-Gardner A. The Role of Serum Haemagglutination-Inhibiting Antibody in Protection Against Challenge Infection With Influenza A2 and B Viruses. J Hygiene (1972) 70(4):767–77. doi: 10.1017/s0022172400022610 PMC2130285 4509641

[15] Veguilla V Hancock K Schiffer J Gargiullo P Lu X Aranio D. Sensitivity and Specificity of Serologic Assays for Detection of Human Infection With 2009 Pandemic H1N1 Virus in U.S. Populations. J Clin Microbiol (2011) 49(6):2210–5. doi: 10.1128/JCM.00229-11 PMC3122722 21471339

[16] Kuster SP Shah PS Coleman BL Lam PP Tong A Wormsbecker A. Incidence of Influenza in Healthy Adults and Healthcare Workers: A Systematic Review and Meta-Analysis. PloS One (2011) 6(10):e26239. doi: 10.1371/journal.pone.0026239 22028840 PMC3196543

[17] Jorgensen P Mereckiene J Cotter S Johansen K Tsolova S Brown C. How Close Are Countries of the WHO European Region to Achieving the Goal of Vaccinating 75% of Key Risk Groups Against Influenza? Results From National Surveys on Seasonal Influenza Vaccination Programmes, 2008/2009 to 2014/2015. Vaccine (2018) 36(4):442–52. doi: 10.1016/j.vaccine.2017.12.019 PMC5777640 29287683

[18] Sanyal M Holmes TH Maecker HT Albrecht RA Dekker CL He XS. Diminished B-Cell Response After Repeat Influenza Vaccination. J Infect Dis (2019) 219:10 1586–1595. doi: 10.1093/infdis/jiy685 PMC6473172 30496437

[19] Leung KYV Carolan LA Worth LJ Harper SA Peck H Tilmanis D. Influenza Vaccination Responses: Evaluating Impact of Repeat Vaccination Among Health Care Workers. Vaccine (2017) 35;19:2558–68. doi: 10.1016/j.vaccine.2017.03.063 28385605

[20] Strengell M Ikonen N Ziegler T Kantele A Anttila VJ Julkunen I. Antibody Responses Against Influenza A(H1N1)pdm09 Virus After Sequential Vaccination With Pandemic and Seasonal Influenza Vaccines in Finnish Healthcare Professionals. Influenza Other Respir Viruses (2013) 7(3):431–8. doi: 10.1111/j.1750-2659.2012.00415.x PMC5779819 22913369

[21] Künzel W Glathe H Engelmann H Van Hoecke C. Kinetics of Humoral Antibody Response to Trivalent Inactivated Split Influenza Vaccine in Subjects Previously Vaccinated or Vaccinated for the First Time. Vaccine (1996) 14(12):1108–10. doi: 10.1016/0264-410x(96)00061-8 8911005

[22] Hauge SH Bakken IJ de Blasio BF Håberg SE. Burden of Medically Attended Influenza in Norway 2008-2017. Influenza Other Respi Viruses (2019) 13:240–7. doi: 10.1111/irv.12627 PMC6468058 30637942

[23] van der Most RG Clement F Willekens J Dewé W Walravens K Vaughn DW. Long-Term Persistence of Cell-Mediated and Humoral Responses to A(H1N1)pdm09 Influenza Virus Vaccines and the Role of the AS03 Adjuvant System in Adults During Two Randomized Controlled Trials. Clin Vaccine Immunol (2017) 24:e00553–16. doi: 10.1128/CVI.00553-16 PMC5461372 28446441

[24] Galson JD Trück J Kelly DF van der Most R. Investigating the Effect of AS03 Adjuvant on the Plasma Cell Repertoire Following Ph1n1 Influenza Vaccination. Sci Rep (2016) 6:37229. doi: 10.1038/srep37229 27849037 PMC5110968

[25] Cohet C van der Most R Bauchau V Bekkat-Berkani R Doherty TM Schuind A. Safety of AS03-Adjuvanted Influenza Vaccines: A Review of the Evidence. Vaccine (2019) 37(23):3006–21. doi: 10.1016/j.vaccine.2019.04.048 31031030

[26] Ferguson M Risi G Davis M Sheldon E Baron M Li P. Safety and Long-Term Humoral Immune Response in Adults After Vaccination With an H1N1 2009 Pandemic Influenza Vaccine With or Without AS03 Adjuvant. J Infect Dis (2012) 205(5):733–44. doi: 10.1093/infdis/jir641 22315336

[27] Gaglani M Spencer S Ball S Song J Naleway A Henkle E. Antibody Response to Influenza A(H1N1)pdm09 Among Healthcare Personnel Receiving Trivalent Inactivated Vaccine: Effect of Prior Monovalent Inactivated Vaccine. J Infect Dis (20142014) 209(11):1705–14. doi: 10.1093/infdis/jit825 PMC7313942 24363436

[28] Kim SS Flannery B Foppa IM Chung JR Nowalk MP Zimmerman RK. Effects of Prior Season Vaccination on Current Season Vaccine Effectiveness in the United States Flu Vaccine Effectiveness Network, 2012-2013 Through 2017-2018. Clin Infect Dis (2021) 73(3):497–505. doi: 10.1093/cid/ciaa706 32505128 PMC8326585

[29] Linderman SL Ellebedy AH Davis C Eberhardt CS Antia R Ahmed R. Influenza Immunization in the Context of Preexisting Immunity. Cold Spring Harb Perspect Med (2020) 11(11):a040964. doi: 10.1101/cshperspect.a040964 PMC8559541 32988981

[30] Ellebedy AH. Immunizing the Immune: Can We Overcome Influenza’s Most Formidable Challenge? Vaccines (Basel) (2018) 6(4):68. doi: 10.3390/vaccines6040068 PMC6313899 30248996

[31] Zhang Y Garcia-Ibanez L Toellner KM. Regulation of Germinal Center B-Cell Differentiation [Published Correction Appears in Immunol Rev. 2016 Jul;272(1):202]. Immunol Rev (2016) 270(1):8–19. doi: 10.1111/imr.12396 26864101 PMC4755139

[32] Sherman AC Lai L Bower M Natrajan MS Huerta C Karmali V. The Effects of Imprinting and Repeated Seasonal Influenza Vaccination on Adaptive Immunity After Influenza Vaccination. Vaccines (2020) 8(4):663. doi: 10.3390/vaccines8040663 PMC7712189 33171854

[33] Francis T. On the Doctrine of Original Antigenic Sin. Proc Am Philos Soc (1960) 104:572–8.

[34] Andrews SF Huang Y Kaur K Popova LI Ho IY Pauli NT. Immune History Profoundly Affects Broadly Protective B Cell Responses to Influenza. Sci Transl Med (2015) 7(316):316ra192. doi: 10.1126/scitranslmed.aad0522 PMC4770855 26631631

[35] Madsen A Jul-Larsen Å Trieu MC Krammer F. Cox. Persistently High Antibody Responses After AS03-Adjuvanted H1N1pdm09 Vaccine: Dissecting the HA Specific Antibody Response. NPJ Vaccines (2021) 6:45. doi: 10.1038/s41541-021-00308-5 33795694 PMC8016826

[36] Herrera MT Gonzalez Y Juárez E Hernández-Sánchez F Carranza C Sarabia C. Humoral and Cellular Responses to a Non-Adjuvanted Monovalent H1N1 Pandemic Influenza Vaccine in Hospital Employees. BMC Infect Dis (2013) 13:544. doi: 10.1186/1471-2334-13-544 24238117 PMC3835617

