# Supplementary material for: Functional and Binding H1N1pdm09-Specific Antibody Responses in Occasionally and Repeatedly Vaccinated Healthcare Workers: A Five-Year Study (2009-2014)
Source: Front Immunol. 2021 Dec 6;12:748281. doi: 10.3389/fimmu.2021.748281 (PMC8685392; doi:10.3389/fimmu.2021.748281)
Supplement: Supplementary file 1 [file DataSheet_1.docx]

**Supplementary material**


 **Supplementary Figure 1: The antibody response in HCW from 2009/10 – 2013/14 receiving only the pandemic vaccination.** The ELISA IgG (A), HI (B) and MN titre (C) in HCW pre-pandemic vaccination (D0) and post-pandemic vaccination (day 21, 3, 6, 12 months, 1, 2, 3, 4 years in ELISA and HI, day 21, 12 months, 1, 2, 3, 4 years in MN).

Each datapoint represent a single individual, and the horizontal line indicates the

geometric mean titre with 95 % confidence interval. The dotted line at 40 (B) and 80

(C) represents the protective titre. The Friedman test was used for detecting

differences between the timepoints, with Dunn´s test for multiple comparisons. D0

2009 was used as the reference timepoint. *** P<0.001, ** P<0.01,* P<0.05.


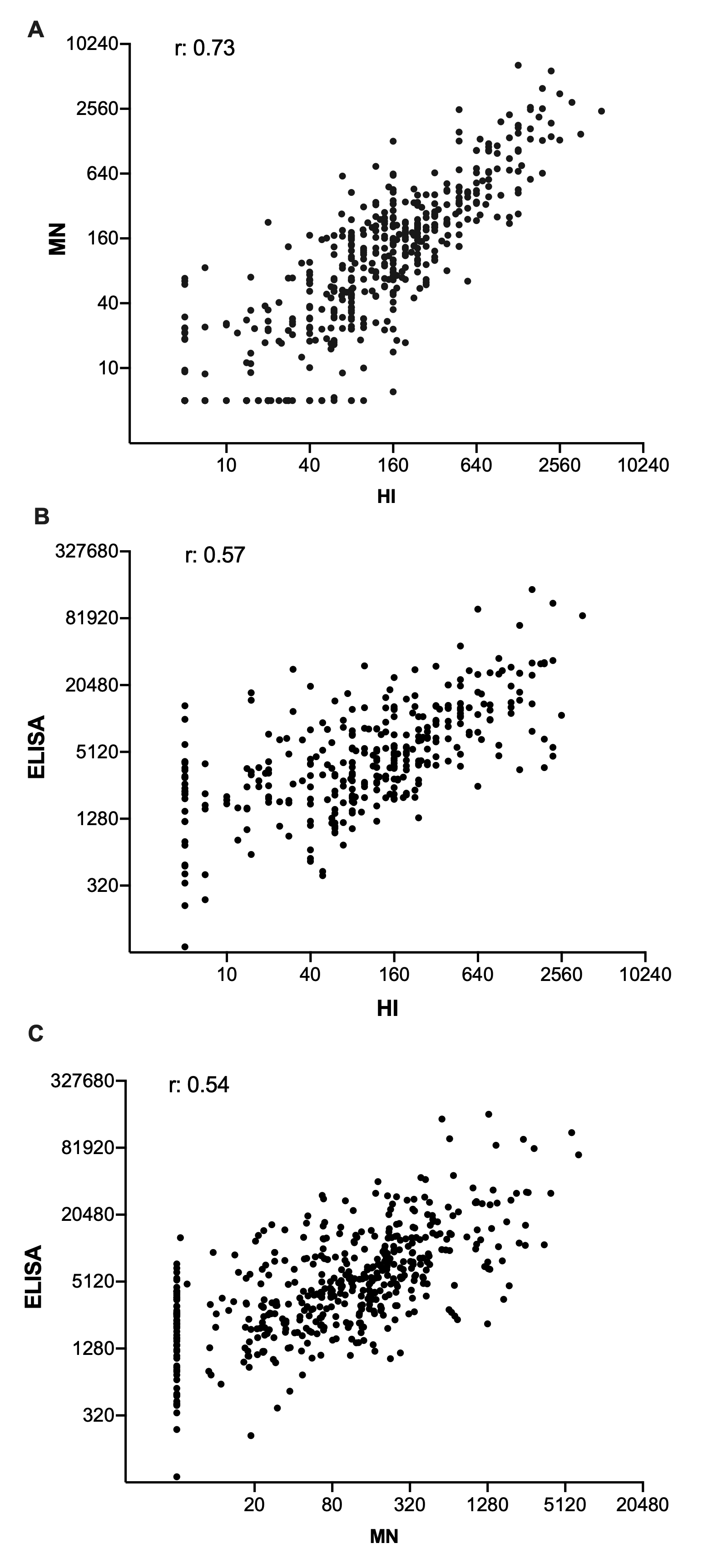


**Figure 2: Correlation between the antibody titres measured by the HI, MN and**

**ELISA assays.** Correlation between MN-HI titres, ELISA-HI and ELISA-MN titres. Each circle indicates one subjects antibody titre at one time point in each assay. The Pearson correlation coefficient is shown.
